# Observational Cohort Study of Evolving Epidemiologic, Clinical, and Virologic Features of Monkeypox in Southern France

**DOI:** 10.3201/eid2812.221440

**Published:** 2022-12

**Authors:** Nadim Cassir, Florian Cardona, Hervé Tissot-Dupont, Christiane Bruel, Barbara Doudier, Salima Lahouel, Karim Bendamardji, Céline Boschi, Sarah Aherfi, Sophie Edouard, Jean-Christophe Lagier, Philippe Colson, Philippe Gautret, Pierre-Edouard Fournier, Philippe Parola, Philippe Brouqui, Bernard La-Scola, Matthieu Million

**Affiliations:** IHU-Méditerranée Infection, Marseille, France (N. Cassir, F. Cardona, H. Tissot-Dupont, B. Doudier, S. Lahouel, K. Bendamardji, C. Boschi, S. Aherfi, S. Edouard, J.-C. Lagier, P. Colson, P. Gautret, P.-E. Fournier, P. Parola, P. Brouqui, B. La-Scola, M. Million);; Aix-Marseille Université, IRD, AP-HM, MEPHI, Marseille (N. Cassir, H. Tissot-Dupont, C. Boschi, S. Aherfi, S. Edouard, J.-C. Lagier, P. Colson, P. Gautret, P. Brouqui, B. La-Scola, M. Million);; Assistance Publique Hôpitaux de Marseille, Marseille (F. Cardona, B. Doudier, S. Lahouel, K. Bendamardji);; Regional Health Agency of Provence-Alpes-Côte d'Azur (ARS Paca), Marseille (C. Bruel);; Aix Marseille Université, IRD, AP-HM, SSA, VITROME, Marseille (P.-E. Fournier, P. Parola)

**Keywords:** monkeypox, viruses, sexually transmitted infections, zoonoses, public health policy, vaccination, emerging communicable diseases, France

## Abstract

We enrolled 136 patients with laboratory-confirmed monkeypox during June 4–August 31, 2022, at the University Hospital Institute Méditerranée Infection in Marseille, France. The median patient age was 36 years (interquartile range 31–42 years). Of 136 patients, 125 (92%) were men who have sex with men, 15 (11%) reported previous smallpox vaccinations, and 21 (15.5%) were HIV-positive. The most frequent lesion locations were the genitals (68 patients, 53%), perianal region (65 patients, 49%), and oral/perioral area (22 patients, 17%). Lesion locations largely corresponded with the route of contamination. Most (68%) patients had isolated anal, genital, or oral lesions when they were first seen, including 56 (61%) who had >1 positive site without a visible lesion. Concurrent sexually transmitted infections were diagnosed in 19 (15%) patients, and 7 patients (5%) were asymptomatic. We recommend vaccination campaigns, intensified testing for sexually transmitted infections, and increased contact tracing to control the ongoing monkeypox outbreak.

Monkeypox virus (MPXV), a zoonotic orthopoxvirus related to smallpox virus, was first described in humans in 1970 in the Democratic Republic of the Congo (*1*). Sporadic outbreaks of infection have been reported in Africa, typically occurring because of close contact with wild rodents, which represent the primary reservoir (*2*). Those outbreaks and travel-associated cases outside of Africa have been characterized by limited secondary spread (*3*). A new outbreak began in May 2022, when autochthonous cases of MPXV infection were initially reported in England and, subsequently, throughout Europe. The World Health Organization declared monkeypox a public health emergency on July 23, 2022 (*4*,*5*). As of September 6, 2022, a total of 54,911 laboratory-confirmed cases of monkeypox and 15 monkeypox-related deaths had been reported to the World Health Organization by 100 countries. Genomic analysis of viral strains causing the current outbreak revealed a distinct phylogenetic lineage of human MPXV. This lineage (B.1, belonging to clade IIb) is characterized by a heightened mutational signature compared with its ancestors and has been associated with milder disease than clade I (*6*).

Initial reports on the ongoing monkeypox outbreak suggest that cases have been atypical; patients have had rashes appearing on fewer regions of the body, frequent genital and perianal lesions, less frequent prodromal symptoms, and a generally benign course of disease (*2*,*7*–*10*). In contrast, in monkeypox-endemic countries in Africa, monkeypox predominantly affects young children and is characterized by rashes incorporating many simultaneous lesions in multiple regions of the body, including the face, that are associated with diffuse lymphadenopathy. The rash is preceded by systemic prodromal symptoms (*3*). Complications include pneumonitis, encephalitis, keratitis, and secondary bacterial infections. The case fatality rate ranges from 1%–10% depending on immune status and clade (*2*). However, these data come from relatively old studies in low resource settings where access to healthcare is limited; minor cases are almost certainly underreported, and ascertainment of cause of death is incomplete.

Detailed information regarding the changing epidemiology of monkeypox during the 2022 outbreak is needed. In this study, we aimed to comprehensively evaluate the epidemiologic, clinical, and virologic features of 136 patients who had a monkeypox diagnosis at the University Hospital Institute Méditerranée Infection in Marseille, France.

## Materials and Methods

### Study Design and Participants

In this retrospective, observational cohort study, we enrolled consecutive patients who had a monkeypox diagnosis during June 4–August 31, 2022, at the University Hospital Institute Méditerranée Infection in Marseille, France. We defined a confirmed monkeypox case as a positive real-time PCR result from skin, genital, rectal, or pharyngeal swab samples. We performed contact tracing and follow-up in collaboration with the regional health agency after mandatory declarations were made to the health agency. Asymptomatic patients were tested when they reported recent high-risk exposure. We only included adult patients in the analysis who had laboratory-confirmed monkeypox. Treatment was prescribed according to national standard of care procedures. Imvanex (Bavarian Nordic, https://www.bavarian-nordic.com) was the vaccine used in France for the preexposure and postexposure vaccination against MPXV.

### Ethics Approval

The study was approved by the ethics committee at the University Hospital Institute Méditerranée Infection (no. 039-2022) and declared to the Règlement Général de la Protection des Données registry (no. 22-340). We extracted medical data from the hospital’s electronic medical records system. We obtained oral informed consent from all patients in accordance with the Declaration of Helsinki (revised in 2013) and written informed consent for publication of clinical images when needed.

### Laboratory Procedures

Laboratory confirmation of orthopoxvirus, including monkeypox virus, was performed at the University Hospital Institute Méditerranée Infection in Marseille, France. We tested skin, genital, rectal, and pharyngeal swabs for MPXV by using in-house real-time PCR (*11*). Genital or rectal swab refers to sampling of skin/mucosal lesions in the genital or rectal areas. No anoscopies were performed in this cohort. For 127 patients who were enrolled after being informed, we tested clinical samples for other sexually transmitted infections (STI) by using quantitative PCR to detect specific pathogens, including *Neisseria gonorrhoeae*, *Chlamydia trachomatis*, *Mycoplasma genitalium*, *Treponema pallidum*, herpesvirus, and *Trichomonas vaginalis* as previously described (*12*,*13*). Using serologic methods, we also tested for hepatitis B virus, hepatitis C virus, HIV, and syphilis in 9 of those 127 patients. All patients were examined in an isolation room where samples were collected for analysis. Sample collection to diagnose monkeypox and other STIs evolved during the time of the study, focusing first on the rash lesion and then associated systematically with pharyngeal, rectal, and genital swabbing together with blood samples for serologies contingent upon the patient’s agreement. We handled all samples in a Biosafety Level 3 laboratory.

### Data

We established case definitions before beginning data collection. For all patients attending our institute for suspected monkeypox infection, we used a standardized medical questionnaire (in English or French) (Appendix Figures 1, 2) to obtain demographic information; patient’s reported smallpox vaccination; HIV status; epidemiologic data, including exposure to someone with monkeypox, travel, attendance at large gatherings, and risk factors for sexually transmitted infections; sexual practices; symptoms; virologic results at multiple body sites, including analysis of PCR cycle threshold (Ct) values; and co-infection with other sexually transmitted pathogens.

We classified sexual orientation as heterosexual or men who have sex with men (MSM) according to the patient’s declaration. We defined acute proctitis as perianal lesions with rectal pain because there were no cases of rectal pain without lesions. We defined tonsillitis as a sore throat and exanthem as a widespread maculopapular rash.

### Statistical Analysis

We reported continuous variables as medians with interquartile ranges (IQRs), where appropriate, and categorical variables as absolute values and percentages. We compared continuous variables by using Mann-Whitney or Kruskal-Wallis nonparametric tests. All tests were 2-sided with a significance threshold of p<0.05. We performed all analyses by using the statistical package R version 4.0.3 (The R Project for Statistical Computing, https://www.r-project.org). We generated graphs using Prism for Mac version 9.0 (GraphPad, https://www.graphpad.com).

## Results

**Figure 1:**
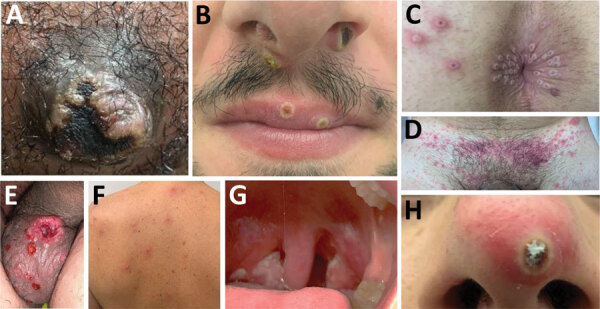
Sites of monkeypox lesions in observational cohort study of evolving epidemiologic, clinical, and virological features of monkeypox in southern France, 2022. A) Primary inoculation site showing an irregular pustule with necrotic crust of the right nipple. B) Pustular lesions with a crusted center on the mucosa of the upper lip, close to the left oral commissure and left nasal orifice. C) Pustules circumferentially distributed on the anal margin and perianal skin of varying sizes and stages of evolution; some with central necrotic crusts. D) Perineally extended purpuric lesions. E) Scrotal lesions of varying sizes and stages of evolution with edema surrounding the larger ulcero-hemorrhagic ulcers. F) Scattered papules, pustules, and umbilicated pustules surrounded by an erythematous halo on the back. G) Reddened and swollen right palatine tonsil with a fibrin-covered ulcer. H) Pustular lesion of the nose with a necrotic central crust, whitish deposit, and erythematous halo.

We enrolled a total of 136 patients who had a laboratory-confirmed monkeypox diagnosis; 133 were men, and 3 were women. We collected demographic (Table 1), clinical (Table 2), and microbiological (Table 3) information for the patients. We determined 125 (92%) patients were MSM and 5 (4%) patients were heterosexual. Information on sexual orientation was not available for the remaining 6 patients. The median age was 36.0 (IQR 30.0–42.0) years. Of the 136 enrolled patients, 15 (11%) reported previous smallpox vaccination, and 21 (16%) were HIV-positive, of whom 5 (24%) had a CD4 cell count of <500 cells/mm^3^. Among 3 heterosexual women, only 1 declared that her regular sexual partner had a monkeypox diagnosis, and the other 2 reported a new sexual partner within the previous 3 weeks. Travel to MPXV-endemic regions was not reported by any patient, whereas 17 (12.5%) reported recent travel to a country that is part of the current outbreak, which includes Spain (n = 11 patients), United States (n = 2 patients), Germany (n = 1 patient), Belgium (n = 1 patient), Canada (n = 1 patient), and Italy (n = 1 patient). Seven (5.2%) patients had attended a Pride event in the previous 21 days in Spain (n = 2) and France (n = 5). The most frequent lesions were located in the genital (68 [53%] patients), perianal (65 [49%] patients), and oral/perioral (22 [17%] patients) areas (Figure 1). The number of skin lesions was <10 in 98 (73%) patients. An asynchronous rash was observed in 41 (30%) of 129 symptomatic patients. Localized lymphadenopathy in lesion areas was observed in 47 (36.4%) patients. Systemic manifestations before or when the patient was first seen included fever (72 [56%] patients), influenza-like illness (44 [35%] patients), and sore throat (11 [8.5%] patients), which preceded the rash in 90 (70%) patients.

**Table 1:** Demographic and epidemiologic characteristics of patients with monkeypox in Marseille during the 2022 outbreak in southern France*

Variable	No. patients†
Age, y, median (IQR)	36 (30–42)
Sex	
F	3 (2.2)
M	133 (97.8)
Sexual orientation	
Men who have sex with men	125 (91.9)
Using PrEP	30 (24)
Heterosexual men	2 (1.5)
Heterosexual women	3 (2.2)
History of smallpox vaccination	
Childhood vaccine	7 (5.1)
Postexposure vaccine	6 (4.4)
Preexposure vaccine	2 (1.5)
HIV-positive	
Total	21 (15.4)
<500 CD4/mm^3^	5 (23.8)
<200 CD4/mm^3^	0
Possible exposure to monkeypox	
Sexual partner with monkeypox	21 (15.4)
New sexual partners‡	115 (84.6)
Attendance at a Pride event	7 (5.2)
Recent travel to an endemic country	0
Recent travel to an epidemic country	17 (12.5)

*Values are no. (%) patients except as indicated. PrEP, preexposure prophylaxis; CD4, CD4 T lymphocyte. †Total number of patients in the study was 136. ‡Single or multiple new sexual partners in the previous 21 d.

**Table 2:** Clinical characteristics of symptomatic patients with monkeypox in Marseille during the 2022 outbreak in southern France*

Variable	No. (%) patients
Systemic features	
>1 systemic feature	98 (73)
Systemic symptoms before rash onset	90 (69.8)
Fever	72 (55.8)
Influenza-like illness	44 (35.1)
Sore throat	11 (8.5)
Clinical features of rash	
Approximate number of lesions	
>50	0
11–50	17 (13.2)
1–10	98 (73)
Type of lesion	
Papular	26 (21)
Vesicular	60 (49)
Pustular	25 (20)
Scabbed	10 (7.8)
Asynchronous rash	41 (30.1)
Lesion location	
Genital	68 (52.7)
Perianal	63 (48.8)
Oral ulcer	3 (2.3)
Perioral	22 (17.1)
Hands and feet	13 (10.1)
Trunk	28 (21.7)
Lymphadenopathy	
Any lymphadenopathy	51 (39.5)
Regional at site of lesion	47 (36.4)
Cervical	26 (20.2)
Inguinal	28 (21.7)
Generalized	4 (3.1)
Complications	
Any complication	34 (26.4)
Complication by type	
Proctitis	30 (23.3)
Tonsillitis	5 (3.9)
Penile edema	5 (3.9)
Bacterial skin abscess	4 (3.1%)
Exanthem	3 (2.3%)

*Total number of patients evaluated was 129.

**Table 3:** PCR and microbiological results for patients with monkeypox in Marseille during the 2022 outbreak in southern France*

Variable	Positive samples/total (%)	Mean Ct (SD)
PCR results		
Skin swabs	69/84 (82)	28.3 (4.7)
Genital swabs	67/69 (97)	26.2 (4.0)
Throat swabs	50/116 (43)	32.2 (3.4)
Rectal swabs	68/105 (65)	26.1 (5.2)
Concurrent STIs		
HIV	2/9 (22.2)	NA
* Chlamydia trachomatis*	4/127 (3.1)	NA
* Neisseria gonorrhoeae*	13/127 (10.2)	NA
* Mycoplasma genitalium*	1/127 (0.8)	NA
Syphilis	4/127 (3.1)	NA
* Trichomonas vaginalis*	1/127 (0.8)	NA
Other STIs	19/127 (15)	NA

*Ct, cycle threshold; STI, sexually transmitted infection; NA, not applicable.

A concomitant diagnosis of another STI occurred for 19 (15%) patients, including 2 patients who had a new diagnosis of HIV-1 infection. Of 63 patients with perianal lesions, 55 (87.3%) reported practicing receptive anal sex, and 12 (19%) had concomitant *N. gonorrhoeae* (n = 9), *C. trachomatis* (n = 2), or *M. genitalium* (n = 1) infections diagnosed from a rectal swab sample. Of 23 patients with oral ulcers or peri-oral lesions, 6 (26%) had concomitant *N. gonorrhoeae* (n = 4), *C. trachomatis* (n = 1), or *T. vaginalis* (n = 1) infections, and 20 (87%) reported practicing oral-receptive sex. Concomitant syphilis was diagnosed in 1 patient from a genital swab sample, and 3 patients had serologic results indicating active syphilis infections.

We observed complications in 37 (27%) patients, which included proctitis (n = 30), tonsillitis (n = 5), penile edema (n = 5), skin abscesses (n = 5), and exanthem (n = 3). Hospital admission was required for 6 (4.5%) patients, some of whom had multiple issues; 5 required perianal pain relief, 3 required management of bacterial abscesses, 1 had dysphagia because of oral lesions, and 1 was admitted for social reasons. All 6 patients had favorable outcomes. None of the patients received antiviral treatment. Opioid prescription for perianal pain relief was required for 3 hospitalized patients and 2 outpatients.

**Figure 2:**
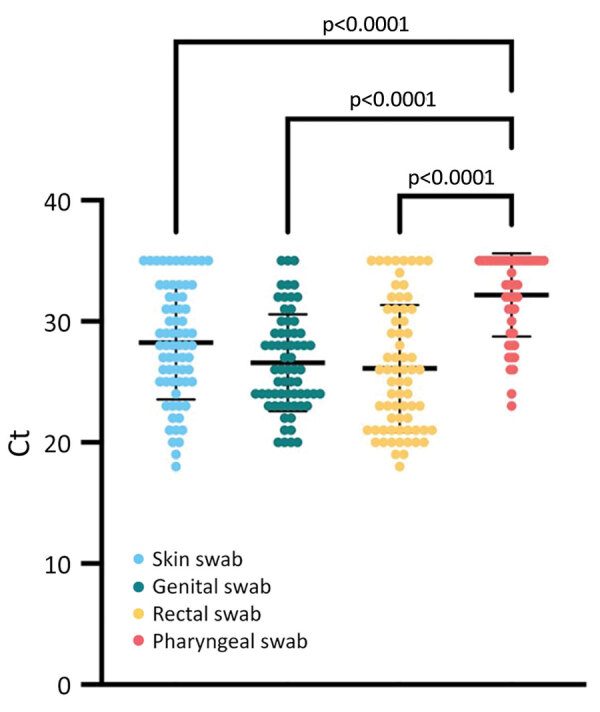
Comparison of monkeypox virus loads between different clinical sampling sites in an observational cohort study of evolving epidemiologic, clinical, and virological features of monkeypox in southern France, 2022. Each colored circle indicates a tested sample; thick horizontal lines indicate mean values; error bars indicate SD. We tested clinical samples for monkeypox virus by using quantitative PCR. We collected samples from skin (n = 69), genital (n = 67), rectal (n = 68), and pharyngeal (n = 50) swabs and calculated the mean Ct for each sample type. Viral loads were significantly lower in skin, genital, and rectal samples compared with pharyngeal samples. Ct, cycle threshold.

We determined the PCR Ct values were lower for skin, genital, and rectal swabs (combined mean) than for pharyngeal specimens (mean Ct ± SD 27.4 ±4.9 vs. 32.2 ±3.4; p<0.0001) (Figure 2). Of 129 symptomatic patients, 15 (12%) had samples taken from 4 sites (pharynx, rectum, skin lesion, genital lesion), 73 (57%) had samples taken from 3 sites, 43 (33%) had samples taken from 2 sites, and 5 (4%) had samples taken from only 1 site (4 skin swabs, 1 genital swab). We observed 92 (68%) patients with isolated anal, genital, skin, or oral lesions when they were first seen, including 56 (61%) patients who had >1 other PCR-positive site without visible lesions. Some PCR-positive samples did not come from visible lesions at the time of testing (40/50 oropharyngeal, 4/67 genital, and 5/68 rectal samples). PCR Ct values from the lesions were not significantly different from those for sites that had no visible lesions (data not shown). The combination of genital and rectal swab testing led to the diagnosis of 111 (86%) MPX cases.

We determined that 7 (5%) patients were asymptomatic; 1 had an MPXV-positive pharyngeal sample and 6 had MPXV-positive rectal samples. We showed that the PCR Ct values obtained from rectal swabs were not significantly different between asymptomatic patients (n = 6) and symptomatic patients (n = 62) (Appendix Figure 3). None of the asymptomatic patients had been previously vaccinated against smallpox virus. We did not find differences in clinical features and MPXV loads between clinical samples from patients who reported receiving smallpox vaccination and those who did not receive vaccination (data not shown) or between patients who reported being HIV-positive and those who did not (Appendix Figure 4).

## Discussion

We report epidemiologic, clinical, and virologic data from 136 patients who had confirmed monkeypox at the outpatient unit of the University Hospital Institute Méditerranée Infection in Marseille, France, during June 4–August 31, 2022. We observed systemic manifestations in approximately two thirds of these patients. Lymphadenopathy often occurred in lesion areas, which differs from that reported for endemic monkeypox in countries of Africa. We observed that the evolution of rashes in our study was also atypical because rashes did not always occur with monomorphic and synchronous vesicular umbilicated lesions (*3*). Similar to recent studies, the cases in our study occurred almost exclusively within the MSM community (*2*,*7*–*10*). However, the percentage of MSM in this study (92%) was lower compared with a previous multicountry report (98%) (*2*). Of note, both male patients in our study who identified as heterosexual had perianal lesions, which raises the question of reporting bias. We also found that most patients had a low number of lesions (<10) located in the genital, anal, and oral regions. Most patients had previous sexual exposure to a person known to have monkeypox or had high risk for sexually transmitted diseases, such as having single or multiple new sexual partners within 21 days before their monkeypox diagnosis or attendance at a Pride event. Similar to results from 3 other large cohorts of patients with monkeypox (*2*,*8*,*9*), we found a high rate (15%) of concurrent STI diagnoses, including 2 patients with a new diagnosis of HIV-1 infection. These findings suggest that the sexual route was the main transmission method for amplification of the monkeypox outbreak. For example, 3 large MSM gatherings have been implicated as monkeypox amplifying and superspreading events in Antwerp, Belgium, and in Madrid and the Canary Islands, Spain (*14*). Although we did not collect this specific information, some patients in our study likely participated in these events. Furthermore, we found that MPXV PCR Ct values in skin, genital, and rectal swab samples were substantially lower than in pharyngeal swab samples. In contrast to a previous report (*15*), we found that MPXV PCR Ct values in genital and rectal swab samples were not significantly lower than those in skin swab samples. These differences might be explained in part by sampling bias; a single site swab might be insufficient for microbiological confirmation of monkeypox. Among clinical samples and independently of the lesion site, we found that genital swab specimens had the highest MPXV positivity rate (97%), followed by skin swab specimens (82%). Rectal swab specimens had a positivity rate of 65% and also contributed to a monkeypox diagnosis. In contrast, the MPXV positivity rate of pharyngeal swab samples was relatively low (43%). Genital and rectal swab specimens together had a remarkably high sensitivity (86%) in this patient cohort, which is noteworthy because these specimens are routinely collected in STI clinics. We also found that some positive samples did not come from areas with visible lesions at the time of testing. Testing specimens from multiple sites on persons with exposure history could, therefore, be relevant depending on the symptoms and potential risks the person had during close contact.

We observed that 15 patients had acquired monkeypox despite having been vaccinated against smallpox during childhood or having received a preexposure or postexposure vaccine. The median period between postexposure vaccination (first dose), and the onset of symptoms was 15 (IQR 8.6–22.4) days. Symptoms occurred at 33 and 35 days in 2 patients after first dose preexposure vaccination. These results warrant further investigation to determine the extent of protection provided by vaccination and highlight the insufficient protection provided by the first dose of vaccine. In addition, 15% of patients were HIV-positive, including 5 patients with CD4 cell counts of <500 cells/mm^3^. Clinical features and MPXV loads did not differ between persons who were HIV-positive and HIV-negative. None of the patients in our study had a CD4 cell count of <200 cells/mm^3^; therefore, we cannot extrapolate our results to immunocompromised patients. Although >25% of patients had complications that required supportive care and antimicrobial drugs for bacterial skin abscesses, we observed a benign course of disease that did not require antiviral therapy. Of note, hospitalized patients did not have conditions that were considered risk factors for a severe form of monkeypox, such as being immunocompromised, a child, or a pregnant or breastfeeding woman. However, to our knowledge, an evaluation of risk factors for severe outcomes during the current outbreak has not been performed. In a recent report on a cohort of 264 monkeypox cases (*7*), 6% of patients were hospitalized exclusively for the management of severe local complications at the site of the rash and for pain relief. All patients were men who were not immunosuppressed. Compassionate use of tecovirimat for the treatment of monkeypox infection has been proposed in some centers (*16*) but requires further evaluation and rational use considering the generally benign course of disease in the current outbreak.

In our study, 7 patients with laboratory-confirmed monkeypox were asymptomatic. In contrast, most large cohort studies during the outbreak have included only symptomatic patients who were positive for MPXV (*2*,*7*–*9*). We tested asymptomatic MSM attending our institute only when they reported recent high-risk exposure, and we likely underestimated the incidence of asymptomatic patients who had laboratory-confirmed monkeypox because this was not part of a systematic screening process. Positive MPXV quantitative PCR results have been identified from anal samples in 13/323 (4%) asymptomatic MSM by assessing the presence of MPXV in anorectal samples from asymptomatic MSM who were routinely tested for STIs (*17*). Moreover, MPXV was identified by PCR in anorectal samples from 3 asymptomatic men in another study; MPXV was cultured from 2 of these samples, indicating viral shedding that could lead to transmission (*18*). These findings suggest that testing and quarantine for only symptomatic persons might be insufficient to contain the current outbreak.

In conclusion, the ongoing human monkeypox outbreak features unusual characteristics, such as the predominant involvement of MSM who have isolated anal, genital, or oral lesions. The high proportion of concomitant STIs showed transmissibility of monkeypox occurred through local inoculations during sexual activity. Testing specimens from multiple sites in persons with relevant exposure history could be relevant, regardless of the presence of visible lesions. Furthermore, asymptomatic carriage of MPXV suggests that some monkeypox cases might remain undiagnosed, and testing and quarantine of only symptomatic patients might be insufficient to contain transmission. Undiagnosed infections might play a role in overall disease transmission during the outbreak among MSM who have a dense sexual network that includes anonymous contacts, which hampers efficient contact tracing. Physicians should be aware that monkeypox symptoms might overlap with those of other STIs, more specifically in MSM with at-risk sexual activity. Prevention, including vaccination campaigns targeting at-risk groups, are ongoing in multiple countries and should be expanded. Intensified testing for all STIs at different sites (pharynx, rectum, genital, and skin) and in urine and increased contact tracing might also be helpful to control the outbreak.

Appendix Additional information for observational cohort study of evolving epidemiologic, clinical, and virologic features of monkeypox in southern France.
