# Supplementary material for: Observational Cohort Study of Evolving Epidemiologic, Clinical, and Virologic Features of Monkeypox in Southern France
Source: Emerg Infect Dis. 2022 Dec;28(12):2409–15. doi: 10.3201/eid2812.221440 (PMC9707593; doi:10.3201/eid2812.221440)
Supplement: Appendix — Additional information for observational cohort study of evolving epidemiologic, clinical, and virologic features of monkeypox in southern France. [file 22-1440-Techapp-s1.pdf]

Article DOI: https://doi.org/10.3201/eid2812.221440

Observational Cohort Study of Evolving Epidemiologic, Clinical, and Virologic Features of Monkeypox in Southern France

Appendix

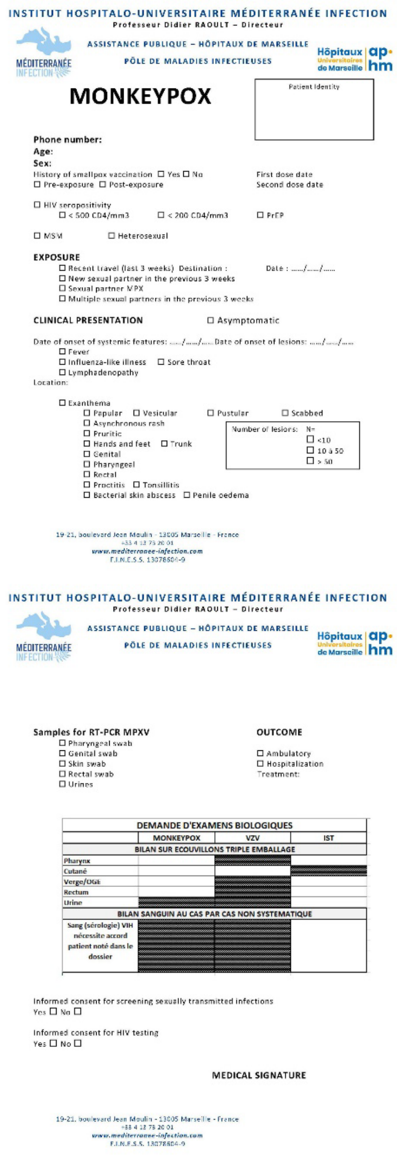

INSTITUT HOSPITALO-UNIVERSITAIRE MÉDITERRANÉE INFECTION
Professeur Didier RAOULT – Directeur
ASSISTANCE PUBLIQUE – HÔPITAUX DE MARSEILLE
PÔLE DE MALADIES INFECTIEUSES
MÉDITERRANÉE INFECTION
Hôpitaux Universitaires de Marseille ap·hm

MONKEYPOX

Patient Identity

Phone number:
Age:
Sex:
History of smallpox vaccination ☐ Yes ☐ No
☐ Pre-exposure ☐ Post-exposure
First dose date
Second dose date

☐ HIV seropositivity
☐ < 500 CD4/mm3 ☐ < 200 CD4/mm3 ☐ PrEP

☐ MSM ☐ Heterosexual

EXPOSURE
☐ Recent travel (last 3 weeks) Destination : Date : ……/……/……
☐ New sexual partner in the previous 3 weeks
☐ Sexual partner MPX
☐ Multiple sexual partners in the previous 3 weeks

CLINICAL PRESENTATION ☐ Asymptomatic

Date of onset of systemic features: ……/……/…… Date of onset of lesions: ……/……/……
☐ Fever
☐ Influenza-like illness ☐ Sore throat
☐ Lymphadenopathy
Location:

☐ Exanthema
☐ Papular ☐ Vesicular ☐ Pustular ☐ Scabbed
☐ Asynchronous rash
☐ Pruritic
☐ Hands and feet ☐ Trunk
☐ Genital
☐ Pharyngeal
☐ Rectal
☐ Proctitis ☐ Tonsillitis
☐ Bacterial skin abscess ☐ Penile oedema

Number of lesions: N=
☐ <10
☐ 10 à 50
☐ > 50

19-21, boulevard Jean Moulin - 13005 Marseille - France
+33 4 13 73 20 01
www.mediterranee-infection.com
F.I.N.E.S.S. 13078604-9

INSTITUT HOSPITALO-UNIVERSITAIRE MÉDITERRANÉE INFECTION
Professeur Didier RAOULT – Directeur
ASSISTANCE PUBLIQUE – HÔPITAUX DE MARSEILLE
PÔLE DE MALADIES INFECTIEUSES
MÉDITERRANÉE INFECTION
Hôpitaux Universitaires de Marseille ap·hm

Samples for RT-PCR MPXV
☐ Pharyngeal swab
☐ Genital swab
☐ Skin swab
☐ Rectal swab
☐ Urines

OUTCOME
☐ Ambulatory
☐ Hospitalization
Treatment:

DEMANDE D'EXAMENS BIOLOGIQUES			
	MONKEYPOX	VZV	IST
BILAN SUR ECOUVILLONS TRIPLE EMBALLAGE			
Pharynx			
Cutané			
Verge/OGE			
Rectum			
Urine			
BILAN SANGUIN AU CAS PAR CAS NON SYSTEMATIQUE			
Sang (sérologie) VIH nécessite accord patient noté dans le dossier			

Informed consent for screening sexually transmitted infections
Yes ☐ No ☐

Informed consent for HIV testing
Yes ☐ No ☐

MEDICAL SIGNATURE

19-21, boulevard Jean Moulin - 13005 Marseille - France
+33 4 13 73 20 01
www.mediterranee-infection.com
F.I.N.E.S.S. 13078604-9

Appendix Figure 1. Standardized medical questionnaire (in English) used for observational cohort study of evolving epidemiologic, clinical, and virological features of monkeypox in southern France. We used

the questionnaire to obtain demographic information; patient-reported smallpox vaccination status; HIV status; epidemiologic data, including exposure to someone with monkeypox, travel, attendance at large gatherings, and risk factors for sexually transmitted infections; sexual practices; symptoms; virological results at multiple body sites, including analysis of PCR cycle threshold (Ct) values; and co-infection with other sexually transmitted pathogens.

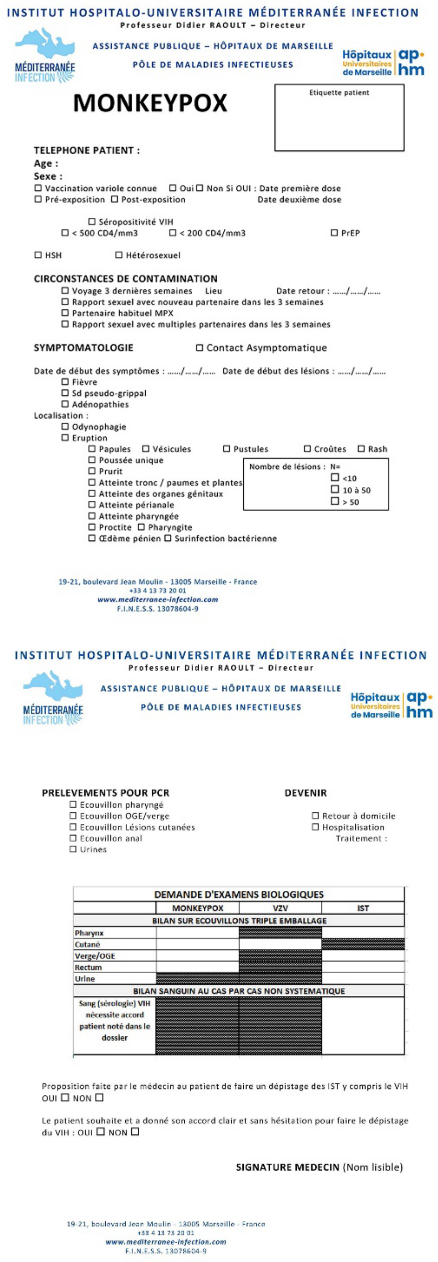

INSTITUT HOSPITALO-UNIVERSITAIRE MÉDITERRANÉE INFECTION
Professeur Didier RAOULT – Directeur
ASSISTANCE PUBLIQUE – HÔPITAUX DE MARSEILLE
PÔLE DE MALADIES INFECTIEUSES
MÉDITERRANÉE INFECTION
Hôpitaux Universitaires de Marseille | ap-hm

MONKEYPOX

Etiquette patient

TELEPHONE PATIENT :
Age :
Sexe :
☐ Vaccination variole connue ☐ Oui ☐ Non Si OUI : Date première dose
☐ Pré-exposition ☐ Post-exposition Date deuxième dose

☐ Séropositivité VIH
☐ < 500 CD4/mm3 ☐ < 200 CD4/mm3 ☐ PrEP

☐ HSH ☐ Hétérosexuel

CIRCONSTANCES DE CONTAMINATION
☐ Voyage 3 dernières semaines Lieu Date retour : ……/……/……
☐ Rapport sexuel avec nouveau partenaire dans les 3 semaines
☐ Partenaire habituel MPX
☐ Rapport sexuel avec multiples partenaires dans les 3 semaines

SYMPTOMATOLOGIE ☐ Contact Asymptomatique

Date de début des symptômes : ……/……/…… Date de début des lésions : ……/……/……
☐ Fièvre
☐ Sd pseudo-grippal
☐ Adénopathies
Localisation :
☐ Odynophagie
☐ Eruption
☐ Papules ☐ Vésicules ☐ Pustules ☐ Croûtes ☐ Rash
☐ Poussée unique
☐ Prurit
☐ Atteinte tronc / paumes et plantes
☐ Atteinte des organes génitaux
☐ Atteinte périanale
☐ Atteinte pharyngée
☐ Proctite ☐ Pharyngite
☐ Œdème pénien ☐ Surinfection bactérienne

Nombre de lésions : N=
☐ <10
☐ 10 à 50
☐ > 50

19-21, boulevard Jean Moulin - 13005 Marseille - France
+33 4 13 73 20 01
www.mediterranee-infection.com
F.I.N.E.S.S. 13078604-9

INSTITUT HOSPITALO-UNIVERSITAIRE MÉDITERRANÉE INFECTION
Professeur Didier RAOULT – Directeur
ASSISTANCE PUBLIQUE – HÔPITAUX DE MARSEILLE
PÔLE DE MALADIES INFECTIEUSES
MÉDITERRANÉE INFECTION
Hôpitaux Universitaires de Marseille | ap-hm

PRELEVEMENTS POUR PCR
☐ Ecouvillon pharyngé
☐ Ecouvillon OGE/verge
☐ Ecouvillon Lésions cutanées
☐ Ecouvillon anal
☐ Urines

DEVENIR
☐ Retour à domicile
☐ Hospitalisation
Traitement :

DEMANDE D'EXAMENS BIOLOGIQUES			
	MONKEYPOX	VZV	IST
BILAN SUR ECOUVILLONS TRIPLE EMBALLAGE			
Pharynx			
Cutané			
Verge/OGE			
Rectum			
Urine			
BILAN SANGUIN AU CAS PAR CAS NON SYSTEMATIQUE			
Sang (sérologie) VIH nécessite accord patient noté dans le dossier			

Proposition faite par le médecin au patient de faire un dépistage des IST y compris le VIH
OUI ☐ NON ☐

Le patient souhaite et a donné son accord clair et sans hésitation pour faire le dépistage du VIH : OUI ☐ NON ☐

SIGNATURE MEDECIN (Nom lisible)

19-21, boulevard Jean Moulin - 13005 Marseille - France
+33 4 13 73 20 01
www.mediterranee-infection.com
F.I.N.E.S.S. 13078604-9

Appendix Figure 2. Standardized medical questionnaire (in French) used for observational cohort study of evolving epidemiologic, clinical, and virological features of monkeypox in southern France. We used the questionnaire to obtain demographic information; patient-reported smallpox vaccination status; HIV

status; epidemiologic data, including exposure to someone with monkeypox, travel, attendance at large gatherings, and risk factors for sexually transmitted infections; sexual practices; symptoms; virological results at multiple body sites, including analysis of PCR cycle threshold (Ct) values; and co-infection with other sexually transmitted pathogens.

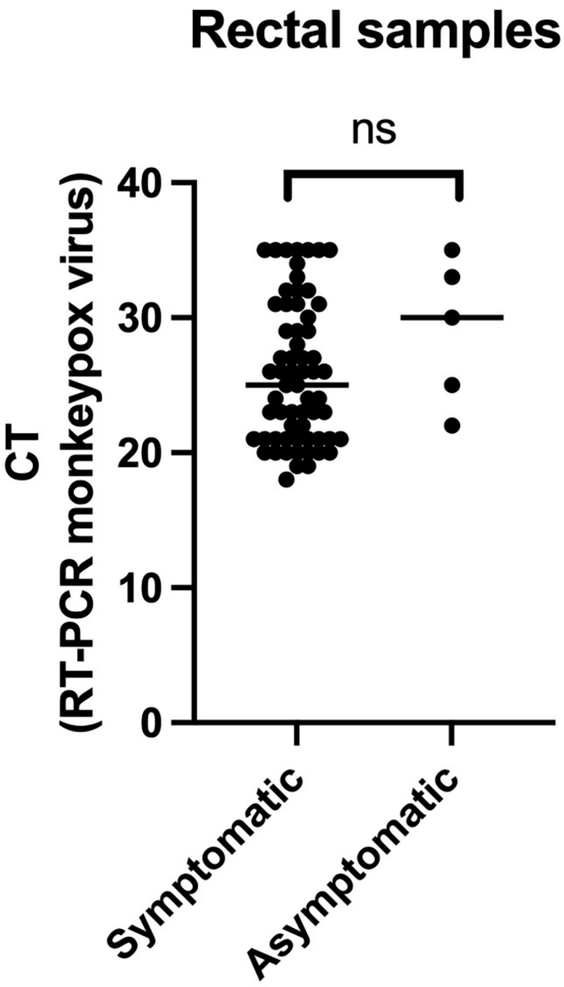

Appendix Figure 3. Comparison of monkeypox virus loads in rectal samples from symptomatic and asymptomatic patients in Marseille in an observational cohort study of evolving epidemiologic, clinical, and virological features of monkeypox in southern France, 2022. We tested rectal swabs from symptomatic (n = 62) and asymptomatic (n = 6) patients for monkeypox virus by using quantitative PCR and calculated the mean Ct for each group. We found no difference in viral loads between the groups. Ct, cycle threshold; ns, not significant.

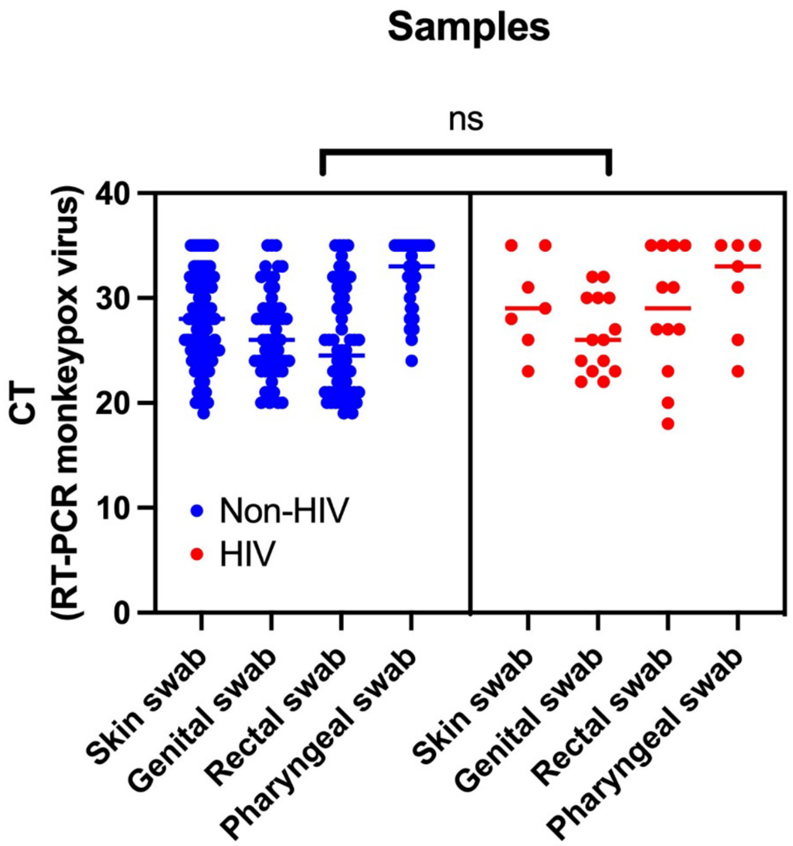

Appendix Figure 4. Comparison of monkeypox virus loads in clinical samples from patients with HIV and those without HIV infections in Marseille in an observational cohort study of evolving epidemiologic, clinical, and virological features of monkeypox in southern France, 2022. We tested clinical samples for monkeypox virus by using quantitative PCR. We collected samples from skin (n = 69), genital (n = 67), rectal (n = 68), and pharyngeal (n = 50) swabs and calculated the mean Ct for each sample type. We found no differences in viral loads between the groups. Ct, cycle threshold; ns, not significant.
